# Causes of hospital admission and mortality among 6683 people who use heroin: A cohort study comparing relative and absolute risks

**DOI:** 10.1016/j.drugalcdep.2019.06.027

**Published:** 2019-11-01

**Authors:** Dan Lewer, Emily J. Tweed, Robert W. Aldridge, Katherine I. Morley

**Affiliations:** a Collaborative Centre for Inclusion Health, University College London, 1-19 Torrington Place, London WC1E 7HB, UK; b National Addictions Centre, Institute of Psychiatry, Psychology & Neuroscience, King’s College London, 4 Windsor Walk, Camberwell, London SE5 8AF, UK; c Institute of Health Informatics, University College London, 222 Euston Road London, NW1 2DA, UK; d South London and Maudsley NHS Foundation Trust, Bethlem Royal Hospital, Monks Orchard Road, Beckenham BR3 3BX, UK; e MRC/CSO Social and Public Health Sciences Unit, University of Glasgow, Top Floor, 200 Renfield Street, Glasgow, G2 3AX, UK; f RAND Europe, Westbrook Centre, Milton Road, Cambridge, CB4 1YG, UK

**Keywords:** Heroin, Crack cocaine, Substance-related disorders, Public health, Epidemiology

## Abstract

**Background:**

Mortality in high-risk groups such as people who use illicit drugs is often expressed in relative terms such as standardised ratios. These measures are highest for diseases that are rare in the general population, such as hepatitis C, and may understate the importance of common long-term conditions.

**Population:**

6683 people in community-based treatment for heroin dependence between 2006 and 2017 in London, England, linked to national hospital and mortality databases with 55,683 years of follow-up.

**Method:**

Age- and sex-specific mortality and hospital admission rates in the general population of London were used to calculate the number of expected events. We compared standardised ratios (relative risk) to excess deaths and admissions (absolute risk) across ICD-10 chapters and subcategories.

**Results:**

Drug-related diseases had the highest relative risks, with a standardised mortality ratio (SMR) of 48 (95% CI 42–54) and standardised admission ratio (SAR) of 293 (95% CI 282–304). By contrast, other diseases had an SMR of 4.4 (95% CI 4.0–4.9) and an SAR of 3.15 (95% CI 3.11–3.19). However, the majority of the 621 excess deaths (95% CI 569–676) were not drug-related (361; 58%). The largest groups were liver disease (75 excess deaths) and COPD (45). Similarly, 80% (11,790) of the 14,668 excess admissions (95% CI 14,382–14,957) were not drug-related. The largest groups were skin infections (1073 excess admissions), alcohol (1060), COPD (812) and head injury (612).

**Conclusions:**

Although relative risks of drug-related diseases are very high, most excess morbidity and mortality in this cohort was caused by common long-term conditions.

## Highlights

• People who use heroin have very high risk of hospitalisation and death.
• Most of the excess risk relates to common non-communicable diseases.
• Diseases of the lung, cardiovascular system and liver cause large numbers of deaths.

## 1 Introduction

People who use illicit opioids such as heroin have high rates of mortality and morbidity, with an age-standardised mortality risk of between four and 15 times the general population (Degenhardt et al., 2011). In England, people identified as dependent on opiates by drug treatment and criminal justice services have a six-fold increased risk of death (Pierce et al., 2015). There are fewer studies of morbidity in this population, with most focusing on blood borne viral infections and mental health problems (Aldridge et al., 2018).

Although drug overdose is often found to be the largest single cause of death among people who are dependent on illicit drugs, it usually accounts for less than half of deaths overall (Degenhardt et al., 2011; Gao et al., 2019; Pierce et al., 2015). Available data suggests cardiovascular disease, respiratory disease, and liver disease also cause many deaths among people who use drugs, and account for an increasing proportion as age increases (Degenhardt et al., 2014; Merrall et al., 2013; Pierce et al., 2015). Although these conditions are common in the general population, their contribution to excess morbidity and mortality among people who use illicit drugs is unclear.

People who are dependent on illicit drugs have extremely high relative risks of overdose, hepatitis C, HIV and mental health problems. Some diseases have become specifically associated with illicit drug use, including hepatitis C in many high income countries. Treatment and harm reduction initiatives to improve health among people who use drugs have focused on these areas. In contrast, conditions such as cardiovascular disease, which may cause greater absolute morbidity and mortality yet have lower relative risks because they are common in the general population, have received less attention. Due to the frequent disparities between relative and absolute risks in epidemiological studies, the STROBE guidelines for reporting of observational studies recommend reporting both where possible (Vandenbroucke et al., 2014). However, few studies actually do this (King et al., 2012).

To address this, we have used data from a large cohort of people in treatment for heroin dependence to compare relative and absolute risks of hospital admission (as a proxy for morbidity) and death across a range of disease categories, and reported excess morbidity and mortality in this cohort.

## 2 Materials and methods

We used data from the Clinical Records Interactive Search resource at the South London and Maudsley NHS Foundation Trust Biomedical Research Centre. This is a research repository of anonymised records derived from the electronic health record system of a mental healthcare provider in South London, England (Perera et al., 2016). The study population was 6833 patients aged 18–64 entering community-based treatment for heroin dependence between 1 January 2006 and 31 March 2017. Patients were linked using NHS number, date of birth, sex and postcode to inpatient hospital admissions data from the national Hospital Episode Statistics for England database, and to mortality data from the Office for National Statistics. Linkage was conducted by NHS Digital, a public sector statistical agency. Linkage for hospital admissions was conducted until 31 March 2017 and for mortality until 31 January 2019. Some patients have long periods of engagement with the drug treatment service, while other only attend one appointment, but data linkage was available for all patients regardless of their engagement with the service.

To allow calculation of reference rates for the general population, we also accessed hospital admission data for all residents in the healthcare provider’s catchment area of the London Boroughs of Croydon, Lambeth, Lewisham and Southwark, and the number of deaths by age group, sex and cause for all residents of London from the Office for National Statistics. Denominators for the relevant general populations were derived from publicly available population estimates (Office for National Statistics, 2017). The reference data were accessed for 2006–2016 as the closest available match to the study cohort.

For descriptive purposes, we reported drug use according to standard National Drug Treatment and Monitoring System (NDTMS, Public Health England and Department of Health, 2017) forms collected by the service provider. These data are collected for each patient at entry to the service, treatment reviews and at discharge. We identified patients as heroin users where heroin was listed on any NDTMS form collected during the study period. Poly-drug use is common in this cohort and we also reported other drugs that were listed for at least 10% of patients. Where any NDTMS form listed ‘currently injecting’, we classified the patient as ‘reported injecting during the study’.

### 2.1 Disease categories

We used a two-level hierarchy to classify deaths. First, we grouped deaths according to the ICD-10 chapter (World Health Organization, 2016) of the underlying cause of death (such as ‘respiratory diseases’). Chapters with fewer than 10 deaths during the study period were grouped together under ‘other’. Second, we grouped deaths according to three-character subcategories (such as ‘influenza and pneumonia’), again grouping subcategories with fewer than 10 deaths under ‘other’. We used a similar process to group hospital admissions, classifying admissions based on the primary diagnosis and classifying categories with fewer than 250 admissions under ‘other’. For both mortality and hospital admissions, we separated out ‘drug-related’ events, using the Office for National Statistics definition (Office for National Statistics, 2018) (see Supplementary Material). This identified events where the main cause related directly to the use of illicit drugs. In the mortality data, these events are most commonly overdoses, and are more diverse for hospitalisations, including intoxication and drug withdrawal.

### 2.2 Calculation of relative and absolute risks

We calculated reference rates for death and hospital admission in the general population within sex and age groups (18–24, 25–34, 35–44, 45–54 and 55–64) for each disease. We then calculated the expected numbers of events by applying these reference rates to the study population, with time-at-risk calculated for each stratum and accounting for individuals aging during the study. We calculated standardised mortality ratios (SMRs) and standardised admission ratios (SARs) by dividing observed events by expected events (i.e. indirect standardisation). Excess events were calculated as the observed minus the expected events. A formula for calculating the excess events is provided in the Supplementary Material, together with a worked example including analysis code.

We calculated 95% confidence intervals using the exact poisson method (Garwood, 1936). We also used the Kaplan-Meier method to estimate the risk of all-cause death over ten years.

All analyses were conducted using R version 3.5.1.

## 3 Results

### 3.1 Cohort characteristics

Most patients were aged 25–44 and three-quarters were male, reflecting the demographics of patients entering treatment for opiate dependence nationally (Public Health England and Department of Health, 2017). The majority of patients (72%) used crack cocaine as well as heroin. Use of alcohol, cannabis, and benzodiazepines was also recorded for more than 10% of patients. One in three reported injecting during follow-up, though this is likely to be a lower-bound because patients may not want to disclose injecting while they are prescribed opiate substitution therapy. The cohort characteristics are summarised in Table 1.

**Table 1:** Cohort characteristics for 6683 people who use heroin.

Variable	Level	Number of participants (%)
Age at first treatment episode	18–25	552 (8)
	25–34	2259 (34)
	35–44	2568 (38)
	45–54	1099 (16)
	55–64	205 (3)
Sex	Female	1727 (26)
	Male	4956 (74)
Ethnicity	White	5260 (79)
	Black	803 (12)
	Mixed	212 (3)
	Asian	204 (3)
	Other/unknown	204 (3)
Other drugs	Crack cocaine	4813 (72)
	Alcohol	2961 (44)
	Cannabis	1628 (24)
	Benzodiapines	747 (11)
Reported injecting during the study	Yes	2335 (35)
	No	4348 (65)
**Total**		**6683 (100)**

### 3.2 Mortality

During 55,639 years of follow-up (median 9.4, range 0–13), there were 732 deaths, giving a crude all-cause mortality rate of 132/10,000 person-years and an SMR of 6.6 (95% CI 6.1–7.1). There were 621 excess deaths (95% CI 569–676). A Kaplan-Meier estimate suggested that 12.6% (95% CI 11.7%–13.5%) of patients would have died after 10 years of follow-up, with 2800 remaining in the risk set.

**Fig. 1:**
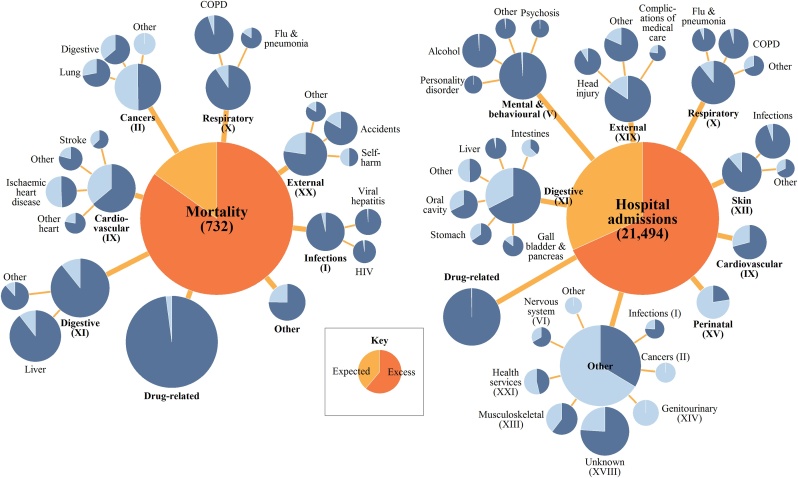
Number of deaths and hospital admissions in a cohort of 6683 people who use heroin in South London, 2016–2019. The area of the circles is proportional to the number of events, while the dark sections show the number of excess events.

Of the 621 excess deaths, 260 (42%) were drug-related and 361 (58%) were due to other causes. The SMRs were 48 (95% CI 42–54) for drug-related deaths and 4.4 (95% CI 4.0–4.9) for all other causes combined. Among ICD-10 chapters, digestive diseases caused the most excess deaths (97), followed by respiratory disease (58) and external causes (46). The largest subcategories were liver disease (75), COPD (45) and accidents (30). The number of deaths and excess deaths are summarised in Fig. 1, with full results are given in Supplementary Material, including comparisons to existing studies.

### 3.3 Hospital admissions

During 44,590 years of follow-up (median 7.7, range 0–11), there were 21,494 hospital admissions, giving a crude admission rate of 482/1,000 person-years and a SAR of 3.15 (95% CI 3.11–3.19). There were 14,668 excess admissions (95% CI 14,382–14,957).

Of the 14,668 excess admissions, 3042 (20.7%) were drug-related and 11,790 (80.3%) were primarily related to other causes. The SARs were 293 (95% CI 282–304) for drug-related admissions and 2.77 (95% CI 2.73–2.81) for all other causes. Among ICD-10 chapters, mental and behavioural disorders caused the most excess admissions (2055), followed by digestive diseases (1940) and external causes (1685). The largest subcategories were mental and behavioural disorders due to alcohol (1060 excess admissions), COPD (812), head injury (612), and diseases of the oral cavity, predominantly dental caries (516).

## 4 Discussion

We conducted a population-based cohort study of people who use heroin and had at least one contact with community-based drug treatment services. Consistent with existing studies of similar populations, our cohort had extreme risk of hospitalisation and death. The majority of excess morbidity and mortality in this population related to common non-communicable diseases.

The use of ‘excess events’ shows the breadth of important diseases in this group, in contrast to the use of relative risks alone, which prioritise drug-related events, blood-borne viruses and mental health problems. The analysis of mortality highlights the importance of digestive, respiratory and cardiovascular diseases, and external causes such as accidents. The analysis of hospital admissions shows that the excess burden of non-communicable diseases is four times greater than for drug-related conditions, and particularly highlights the importance of liver disease, COPD, dental caries and head injuries. Although we provide a more detailed break-down than previous studies, our results are broadly consistent with existing research (Merrall et al., 2013, 2012; Pierce et al., 2015). The literature on health inequalities generally focuses on relative risks, with few studies reporting both relative and absolute risks (King et al., 2012). Our results provide an example of the radically different effects when relative and absolute risks are compared.

We used a linkage system with national mortality and hospital databases, which is important because the study population is likely to be mobile and local databases underestimate the rate of events. A limitation of the analysis is that hospital admissions capture a severe aspect of morbidity, and further research should examine primary care data to provide a more detailed picture. Our data included information on a range of drugs recorded by the drug treatment service, but did not provide detailed information on routes of administration. Three-quarters of patients used both heroin and crack cocaine, but the data does not show whether patients used these drugs separately or mixed them together as ‘speedballs’.

The mortality and hospital admission results showed some different patterns. For example, patients in our cohort were twice as likely to die due to cancer compared to the general population, but were half as likely to be admitted to hospital for cancer treatment. The lower-than-expected rate of hospital admission related to cancer has also been observed for opiate users in Scotland (Merrall et al., 2013). The reasons for this are not clear, but may relate to poorer diagnosis of cancer among people who use heroin, or delayed help-seeking when symptoms occur. Our data also suggest that blood-borne viruses are a common cause of death (5%) but a rare cause of hospitalisation (<1%). This may partly relate to coding practices, as the primary cause of hospitalisation for patients with viral hepatitis or HIV may be listed as something else, such as liver disease or an AIDS-defining illnesses.

The pattern of causes of morbidity and mortality is related to age. In older cohorts of people who use illicit drugs, non-communicable diseases cause larger proportions of deaths (Degenhardt et al., 2014) and relative mortality risks reduce as the reference rates in the general population increase. There may also be secular changes. For example, in cohorts of people who use heroin in Sweden (Nyhlén et al., 2011) and the UK (Oppenheimer et al., 1994) recruited in the 1960s and 70 s and followed-up until the 1990s, more than half of deaths were drug-related. These higher proportions of drug-related mortality may relate to more limited harm reduction services, which were rolled-out in the 1980s and 90 s in many high-income countries. There is some evidence that the average age of people seeking treatment for heroin dependence is increasing (Public Health England and Department of Health, 2017) and the importance of non-communicable diseases is also likely to increase.

Interventions designed to improve the health of people who use heroin have focused on prevention and treatment of blood-borne viral infections and overdoses, as well as reduction in drug-related crime. Needle and syringe programmes, take-home naloxone and opiate substitution are well-researched and effective interventions that target these outcomes (Amato et al., 2005; European Monitoring Centre for and Drugs and Drug Addiction, 2015; Platt et al., 2017). However, there are few interventions that aim to prevent and treat common non-communicable diseases in this group. Our results demonstrate the urgent need for research in this area.

## Ethical approval

The dataset was approved as an anonymised dataset for secondary analyses by the Oxfordshire Research Ethics Committee C (reference number: 08/H0606/71+5). This analysis was approved by the South London and Maudsley NHS Foundation Trust Biomedical Research Centre CRIS Oversight Committee (reference number: 18–122).

## Funding information

DL is funded by the National Institute for Health Research [DRF-2018-11-ST2-016]. This work was supported by a grant from the Wellcome Trust [109823/Z/15/Z] to KIM. EJT is funded by the Medical Research Council (MC_UU_12017/13 and MC_UU_12017/15) and Chief Scientist Office (SPHSU13 and SPHSU15), and by a Chief Scientist Office Clinical Academic Fellowship (CAF/17/11). RWA was supported by the Wellcome Trust through a Clinical Research Career Development Fellowship [206602].

## Contributions

DL conceived of the study. DL, ET, RWA and KIM contributed to the study design. DL conducted data analysis. DL, ET, RWA and KIM drafted and revised the manuscript. All authors have read and approved the final manuscript.

## Declaration of Competing Interest

None.
