# Supplementary material for: Causes of hospital admission and mortality among 6683 people who use heroin: A cohort study comparing relative and absolute risks
Source: Drug Alcohol Depend. 2019 Nov 1;204:107525. doi: 10.1016/j.drugalcdep.2019.06.027 (PMC6891224; doi:10.1016/j.drugalcdep.2019.06.027)
Supplement: Supplementary file 1 [file mmc1.pdf]

Supplementary Information

1. Calculation of excess events
2. Comparison with existing data linkage studies of mortality among opiate users in England and Scotland
3. Comparison with existing data linkage studies of hospital admission among opiate users in Scotland
4. ICD-10 codes for drug-related events
5. References for supplementary material

1 Calculation of excess events

We calculated excess events (excess deaths or excess hospital admissions) using the following formula:

$$observed - \sum_{j=1}^{k} referece_j \times time_j$$

Where 'observed' is the number of events observed in the study cohort, 'reference' is the annual rate of events in the reference population, 'time' is the number of years of follow-up in the study cohort, and 'j' is the demographic stratum (in this case age and sex groups, where k = 10 since our study had 5 age groups and 2 sex groups).

We have posted a version of the analysis code that only requires publicly available data and calculated all-cause excess deaths here: https://osf.io/xbzh8/. This code produces the following table:

		England & Wales, 2006-2016			Study cohort		
Age group	Sex	All-cause deaths	Aggregate population estimate	Reference mortality rate per 100,000 person-years	All-cause deaths*	Years of follow-up	Expected deaths
18-24	Male	15,491	28,752,356	54	17	829	0.4
25-34		31,278	41,385,902	76	115	8,573	6.5
35-44		63,932	42,725,281	150	230	16,308	24.4
45-54		133,208	41,680,133	320	149	12,529	40.0
55-64		282,232	35,193,711	802	44	2,917	23.4
18-24	Female	6,216	28,001,093	22	15	555	0.1
25-34		15,281	41,453,769	37	51	4,311	1.6
35-44		38,316	43,289,966	89	68	5,700	5.0
45-54		90,014	42,485,704	212	38	3,222	6.8
55-64		189,671	36,286,801	523	4	707	3.7
**Total**		**865,639**	**381,254,716**	**227**	**731**	**55,650**	**112.0**

* A random value between -2 and +2 has been added to values in this column to avoid disclosing small counts. The stratum-specific number of deaths in the cohort is provided for interest and are not required for calculation of excess mortality, which uses the total number of deaths in the study cohort.

In this data, the standardised mortality ratio is:

731/112.0 = 6.52 (95% CI 6.06 – 7.02)

While the number of excess deaths is:

731-112.0 = 619 (95% CI 567 – 674)

The confidence intervals are calculated using an exact poisson method (Garwood, 1936), which is also shown in the publicly available code.

2 Comparison with existing data linkage studies of mortality among opiate users in England and Scotland

	**PRESENT STUDY** South London, England 2006-2019 Participants: 6,833 Person-years: 55,639					**Pierce et al., 2015** England 2005-2009 Participants: 198,247 Person-years: 541,891					**Merrall et al., 2012** Scotland 1996-2006 Participants: 32,781 Person-years: 256,275				
ICD-10 group	*Obs*	*CMR*	*Exp*	*Excess*	*SMR*	*Obs*	*CMR*	*Exp*	*Excess*	*SMR*	*Obs*	*CMR*	*Exp*	*Excess*	*SMR*
Total	732	131.6	110.7	621	6.6	3974	73.3	695	3279	5.7	1813	70.7	378.2	1434.8	4.8
Drug-related	266	47.8	5.6	260	47.8	1715	31.6				915	35.7	67.9	847.1	13.5
Non-drug-related	466	83.8	105.2	361	4.4	2259	41.7				898	35	310.3	587.7	2.9
I: Infections	47	8.4	1.7	45	27.8	159	2.9	12.6	146.4	12.6	62	2.4	6.6	55.4	9.4
Viral hepatitis	22	4.0	0.3	22	72.1	82	1.5	1.4	80.6	58.6					
HIV	17	3.1	0.4	17	38.6	31	0.6	4.4	26.6	7					
II: Cancers	69	12.4	34.7	34	2.0	296	5.5	166.3	129.7	1.8	60	2.3	55.6	4.4	1.1
Digestive	28	5.0	10.1	18	2.8	38	0.7	4.1	33.9	9.3					
Lung	25	4.5	7.0	18	3.6										
Other	16	2.9	17.6	-2	0.9										
IX: Cardiovascular	74	13.3	26.8	47	2.8	418	7.7	134.1	283.9	3.1	105	4.1	53.3	51.7	2.0
IHD	31	5.6	15.7	15	2.0										
Other	18	3.2	3.8	14	4.7										
Other heart	14	2.5	3.1	11	4.5										
Stroke	11	2.0	4.1	7	2.7										
X: Respiratory	64	11.5	6.1	58	10.6	259	4.8	29	230	8.9	55	2.1	11.4	43.6	4.8
COPD	48	8.6	2.5	45	19.0	130	2.4	10.4	119.6	12.5					
Flu & Pneumonia	14	2.5	2.2	12	6.3	102	1.9	11.6	90.4	8.8					
XI: Digestive	109	19.6	11.7	97	9.3	423	7.8	65.7	357.3	6.4	147	5.7	32.4	114.6	4.5
Liver	84	15.1	8.8	75	9.6	345	6.4	49.5	295.5	7					
Other	25	4.5	2.9	22	8.6										
XX: External	60	10.8	13.6	46	4.4	482	8.9	146.3	335.7	3.3					
Accidents	36	6.5	6.0	30	6.0										
Other	13	2.3	2.1	11	6.1										
Self-harm	11	2.0	5.5	5	2.0										
Other	43	7.7	10.6	32	4.0										

Obs = observed number of deaths
CMR = crude mortality rate (per 10,000 person-years)
Exp = Expected deaths (based on age- and sex-specific specific admission rates in the general population)
SMR = Standardised mortality ratio
IHD = Ischaemic heart disease
COPD = Chronic obstructive pulmonary disease

3 Comparison with existing data linkage studies of hospital admissions among opiate users in Scotland

	**PRESENT STUDY**<br>South London, England<br>2006-2017<br>Participants: 6,833<br>Person-years: 44,590					**Merrall et al., 2013**<br>Scotland<br>1996-2006<br>Participants: 32,782<br>Person-years: 256,311				
ICD-10 group	*Observed*	*CAR*	*Expected*	*Excess*	*SAR*	*Observed*	*CAR*	*Expected*	*Excess*	*SAR*
Total	21,494	482.0	6,826.0	14,668.0	3.1	75,151	293.2	30,076	45,075	2.5
Drug-related	3,052	68.4	10.4	3,041.6	293.2					
Non-drug-related	18,442	413.6	6,652.2	11,789.8	2.8					
I: Infections	351	7.9	83.8	267.2	4.2	1,928	7.5	521	1,407	3.7
II: Cancers	396	8.9	786.6	-390.6	0.5	1,006	3.9	1,353	-347	0.7
IV: ENM	271	6.1	118.4	152.6	2.3	1,135	4.4	592	543	1.9
V: Mental	2,077	46.6	22.2	2,054.8	93.7	9,046	35.3	*	*	*
Personality disorder	307	6.9	0.9	306.1	342.9					
Other	392	8.8	6.0	386.0	64.8					
Psychosis	305	6.8	2.0	303.0	154.2					
Alcohol	1,073	24.1	13.3	1,059.7	81.0					
VI: Nervous	366	8.2	121.4	244.6	3.0	1,006	3.9	701	305	1.4
IX: Cardiovascular diseases	1,109	24.9	321.3	787.7	3.5	4,312	16.8	1,155	3,157	3.7
X: Respiratory	1,762	39.5	187.6	1,574.4	9.4	3,837	15.0	1,878	1,959	2.0
COPD	847	19.0	35.0	812.0	24.2					
Flu & Pneumonia	519	11.6	30.7	488.3	16.9					
Other	396	8.9	121.9	274.1	3.2					
XI: Digestive	2,873	64.4	933.1	1,939.9	3.1	6,823	26.6	4,323	2,500	1.6
Gall bladder & pancreas	406	9.1	59.5	346.5	6.8					
Intestines	302	6.8	189.8	112.2	1.6					
Liver	423	9.5	15.0	408.0	28.1					
Oral cavity	764	17.1	248.1	515.9	3.1					
Other	524	11.8	263.5	260.5	2.0					
Stomach	454	10.2	157.3	296.7	2.9					
XII: Skin	1460	32.7	165.8	1294.2	8.8	5,041	19.7	1,421	3,620	3.5
Infections	1134	25.4	60.9	1073.1	18.6					
Other	326	7.3	104.9	221.1	3.1					
XIII: Musculoskeletal	942	21.1	373.9	568.1	2.5	2,610	10.2	1,635	975	1.6
Arthropathies	324	7.3	166.9	157.1	1.9					
Other	316	7.1	131.0	185.0	2.4					
Soft tissue	302	6.8	76.0	226.0	4.0					
XIV: Genitourinary	659	14.8	1372.3	-713.3	0.5	2,347	9.2	2,085	262	1.1
XIX: External	2,000	44.9	314.8	1,685.2	6.4					
Head injury	670	15.0	57.6	612.4	11.6					
Medical	253	5.7	57.9	195.1	4.4					
Other	1,077	24.2	199.2	877.8	5.4					
XV-XVI: Perinatal	1,053	23.6	816.3	236.7	1.3					
XVIII: Unknown	2,205	49.5	528.9	1,676.1	4.2					
XXI: Health services	626	14.0	336.6	289.4	1.9					
Other	292	6.5	332.6	-40.6	0.9					

CAR = crude admission rate (per 1,000 person-years)
Exp. = Expected admissions (based on age- and sex-specific specific admission rates in the general population)
SAR = Standardised admission ratio
ENM = Endocrine, nutritional and metabolic (including diabetes)
COPD = Chronic obstructive pulmonary disease
* Could not be calculated because expected admissions are not disaggregated by drug-related events

4 ICD-10 codes for drug-related events

The following codes were used to identify drug-related deaths and hospital admissions. The codes were developed by the Office for National Statistics (Office for National Statistics, 2018).

Description	ICD-10 Codes
Mental and behavioural disorders due to drug use (excluding alcohol and tobacco)	F11–F16, F18–F19
Accidental poisoning by drugs, medicaments and biological substances	X40–X44
Intentional self-poisoning by drugs, medicaments and biological substances	X60–X64
Assault by drugs, medicaments and biological substances	X85
Poisoning by drugs, medicaments and biological substances, undetermined intent	Y10–Y14

5 References for supplementary material

Garwood, F., 1936. Fiducial Limits for the Poisson Distribution. Biometrika 28, 437. https://doi.org/10.2307/2333958

Merrall, E., Bird, S., Hutchinson, S., 2013. A record linkage study of hospital episodes for drug treatment clients in Scotland, 1996–2006. Addict. Res. Theory 21, 52–61. https://doi.org/10.3109/16066359.2012.690052

Merrall, E.L.C., Bird, S.M., Hutchinson, S.J., 2012. Mortality of those who attended drug services in Scotland 1996–2006: Record-linkage study. Int. J. Drug Policy 23, 24–32. https://doi.org/10.1016/j.drugpo.2011.05.010

Office for National Statistics, 2018. Deaths related to drug poisoning in England and Wales: 2017 registrations.

Pierce, M., Bird, S.M., Hickman, M., Millar, T., 2015. National record linkage study of mortality for a large cohort of opioid users ascertained by drug treatment or criminal justice sources in England, 2005–2009. Drug Alcohol Depend. 146, 17–23. https://doi.org/10.1016/j.drugalcdep.2014.09.782
